# Attenuated *Actinobacillus pleuropneumoniae* double-deletion mutant S-8*∆clpP/apxIIC* confers protection against homologous or heterologous strain challenge

**DOI:** 10.1186/s12917-016-0928-9

**Published:** 2017-01-06

**Authors:** Fang Xie, Gang Li, Long Zhou, Yanhe Zhang, Ning Cui, Siguo Liu, Chunlai Wang

**Affiliations:** State Key Laboratory of Veterinary Biotechnology, Division of Bacterial Diseases, Harbin Veterinary Research Institute, Chinese Academy of Agricultural Sciences, Harbin, Heilongjiang People’s Republic of China

**Keywords:** *Actinobacillus pleuropneumoniae*, Live attenuated vaccine, Porcine pleuropneumonia

## Abstract

**Background:**

*Actinobacillus pleuropneumoniae* is the etiological agent of porcine pleuropneumonia, which leads to large economic losses to the swine industry worldwide. In this study, S-8*△clpP△apxIIC*, a double-deletion mutant of *A. pleuropneumoniae* was constructed, and its safety and protective efficacy were evaluated in pigs.

**Results:**

The S-8*△clpP△apxIIC* mutant exhibited attenuated virulence in a murine (BALB/c) model, and caused no detrimental effects on pigs even at a dose of up to 1.0 × 10^9^ CFU. Furthermore, the S-8*△clpP△apxIIC* mutant was able to induce a strong immune response in pigs, which included high levels of IgG1 and IgG2, stimulated gamma interferon (IFN-γ), interleukin 12 (IL-12), and interleukin 4 (IL-4) production, and conferred effective protection against the lethal challenge with *A. pleuropneumoniae* serovars 7 or 5a. The pigs in the S-8*△clpP△apxIIC* immunized groups have no lesions and reduced bacterial loads in the lung tissue after challenge.

**Conclusions:**

The data obtained in this study suggest that the S-8*△clpP△apxIIC* mutant can serve as a highly immunogenic and potential live attenuated vaccine candidate against *A. pleuropneumoniae* infection.

## Background

*Actinobacillus pleuropneumoniae* is a highly adapted pathogen that causes porcine pleuropneumonia, which is an extremely contagious respiratory disease [1]. The disease is often fatal and characterized by fibrinous, hemorrhagic, and necrotic lung lesions, which remains an important global problem in the swine industry [2]. Transmission of the pathogen occurs through an aerosol route during close contact with diseased pigs or asymptomatic carriers, and it can infect pigs of different ages [3]. The clinical features can span from peracute disease with quick death to chronic infection leading to reduced growth rates, and the pigs frequently become asymptomatic carriers. To date, 16 serovars of *A. pleuropneumoniae* have been identified and all serovars can cause disease [4, 5]. Although the incidence of outbreaks has reduced in the developed countries, *A. pleuropneumoniae* remains one of the main causes of economic loss to the global swine production, especially in developing countries [6].

Antimicrobial therapy has been used to prevent and control porcine pleuropneumonia, but it results in the growing problems of multidrug-resistance and antibiotic residues in pigs [7–9]. Concern was raised that multidrug-resistance could be transmitted between different pathogens in pigs followed through the food chain to produce a risk to human health. Thus, vaccination becomes the most effective method of preventing *A. pleuropneumoniae* infection. It has been found that pigs surviving natural infection were protected against homologous and heterologous serovar infection [10]. It is speculated that live bacteria can induce in vivo expression of protective antigens and confer cross-protection. Thus, the application of an attenuated live vaccine is an ideal approach for vaccination against diversified serovars of *A. pleuropneumoniae* [11, 12].

ClpP protease is a family of ATP-dependent protease, which plays a key role in the degradation of misfolded proteins and the stress tolerance in bacteria [13]. The role of ClpP as an important virulence factor has been demonstrated in several pathogenic bacteria [14, 15]. A previous study of *Salmonella typhimurium* and *Salmonella enteritidis* also showed that the virulence of *clpP* deletion mutants were remarkably decreased and that these mutants can serve as live oral vaccine candidates [16]. In our previously study, the *clpP*-deleted mutant of *A. pleuropneumoniae* serovar 7, a prevalent serovar in China, was constructed and its physiological features were analyzed. The ClpP protease mediates *A. pleuropneumoniae* tolerance to multiple environmental stressors, affects the biofilm formation, and may play a critical role in the virulence regulation [17]. The ApxII toxin is the most important virulence factor in *A. pleuropneumoniae* serovar 7, and is encoded by the *apxIICA* gene cluster. The *apxIIA* gene encodes the ApxIIA toxin structural protein, and the *apxIIC* gene encodes the post-translational activating protein that is essential for the ApxII toxin activation, thus disruption in the *apxIIC* gene of *A. pleuropneumoniae* results in secretion of the inactive ApxII toxin but with full antigenicity [18].

In the present study, we constructed the double-deletion mutant S-8*△clpP△apxIIC* of *A. pleuropneumoniae* and evaluated the feasibility of its use as a live attenuated negative marker vaccine based on the virulence, changes in clinical symptoms, immune responses, and protective effects in pigs against challenge with the homologous and heterologous *A. pleuropneumoniae* strains.

## Methods

### Experimental animals

One hundred and ten 6-week-old female BALB/c mice (Beijing Vital River Laboratory Animal Co., Ltd.) were used in the study, with identical feeding conditions. A total of 45 piglets were obtained for use in this study from a farm that was free from *A. pleuropneumoniae* and other respiratory pathogens including *Streptococcus suis*, *Haemophilus parasuis*, and porcine reproductive and respiratory syndrome virus (PCR-negative for nasal and tonsillar swabs and serological-negative in corresponding ELISA assays). The 45 piglets were randomly divided into nine groups of same number and were separately fed with same feeding conditions. The animal experiment in this study was approved by the Animal Ethics Committee of Harbin Veterinary Research Institute of the Chinese Academy of Agricultural Sciences (CAAS) and carried out in strict accordance with animal ethics guidelines and approved protocols.

#### Bacterial strains and growth conditions

*A. pleuropneumoniae* strains S-8 (serovar 7), Shope 4074 (serovar 1), K17 (serovar 5a), the S-8*△clpP* mutant and the S-8*△clpP△apxIIC* mutant were grown at 37 °C in tryptic soy broth (TSB) or tryptic soy agar (TSA) (Becton Dickinson, Franklin Lakes, NJ, USA) containing nicotinamide dinucleotide (NAD, 10 μg/mL; Sigma-Aldrich).

### Chromosomal inactivation of the apxIIC gene of S-8△clpP

Primers IICLF/IICLR, and IICRF/IICRR (Table 1) were used to amplify the two segments flanking with the *apxIIC* gene, IIC-L and IIC-R, as the recombination homologous arms. Using single-overlap extension PCR (SOE PCR), the fragment with a 270 bp internal deletion in the *apxIIC* gene (from nt 18 to 297) was generated, and cloned into the conjugative vector pEMOC2 [19] to construct plasmid pEM*△apxIIC*. Using *E. coli* β2155 and a single-step transconjugation system [20, 21], plasmid pEM*△apxIIC* was applied to introduce the *apxIIC* mutation into the S-8*△clpP* mutant. After two homologous recombination steps, the *A. pleuropneumoniae* S-8*△clpP△apxIIC* mutant strain was verified by PCR and sequencing using IICJDF/IICJDR primers.

**Table 1:** Characteristics of bacterial strains, plasmids, and primers used in this study

Strains, plasmids, and primers	Characteristics or sequence	Source or reference
Strains		
*E. coli* β2155	*thrB1004 pro thi strA hsdS lac*Z△M15 (F’ *lacZ*△M15 *lacl* ^q^ *traD36 proA* ^+^ *proB* ^+^)*△dap* :: *erm* (Erm^r^))*recA* :: *RPA-2-tet*(Tc^r^)::Mu-km (Km^r^) λ*pir*	[21]
*A.pleuropneumoniae* S-8	*A. pleuropneumoniae* serotype 7 clinical isolate from the lung of a diseased pig in northern China	[17]
*A.pleuropneumoniae* S-8*△clpP*	Unmarked *clpP* gene knockout mutant of *A. pleuropneumoniae* S-8	[17]
*A.pleuropneumoniae S-8△clpP△apxIIC*	Unmarked *clpP/apxIIC* genes knockout mutant of *A. pleuropneumoniae* S-8	This work
Plasmids		
pEMOC2	Conjugative vector based on pBluescript SK with mob RP4, polycloning site, *Cm* ^*r*^, and transcriptional fusion of the *omlA* promoter with the *sacB* gene	Accession no. AJ868288 [19]
pEM*△apxIIC*	Conjugative vector pEMOC2 with a 270 bp deletion in the *apxIIC* gene which have a 1.4-kb upstream fragment and 1.4-kb downstream fragment	This work
Primers		
IICLF	5’ GCGTCGACATGACAACACCAATGATTGATTTAC 3’, upstream primer with internal SalI site (underlined)	This work
IICLR	5’ AATCCCCGAA **AGCATCATCC**CTCCCATTC 3’, downstream primer with reverse complement sequence(underlined) of sequence in bold from primer IICRF	This work
IICRF	5’ GGATGATGCT **TTCGGGGATT**CATCTCTATTG 3’, upstream primer with reverse complement sequence(underlined) of sequence in bold from primer IICLR	This work
IICRR	5’ TTGCGGCCGCGTTGTAATAAGTCCCGTAACACCAG 3’, downstream primer with internal NotI site (underlined)	This work
IICJDF	5’ GAAGAGCCATTACCCAACAAC 3’, upstream primer for identification of *apxIIC* gene deletion	This work
IICJDR	5’ ATACAATAGAGATGAATCCCCG 3’, downstream primer for identification of *apxIIC* gene deletion	This work

### Growth experiment and hemolytic assay

*A. pleuropneumoniae* wild-type strain S-8, the S-8*△clpP* mutant, and the S-8*△clpP△apxIIC* mutant were routinely grown in 3 ml of TSB for 16 h, then diluted to OD_600_ of 0.1. The fresh cultures were then inoculated in 30 ml of TSB and grown at 37 °C. The OD_600_ values were recorded at an interval of 1 h using the Eppendorf BioPhotometer (Eppendorf, Germany).

*A. pleuropneumoniae* wild-type strain S-8, the S-8*△clpP* mutant, and the S-8*△clpP△apxIIC* mutant were respectively inoculated onto TSA plates supplemented with 5% defibrinated sheep erythrocytes, and incubated at 37 °C for 18 h. The hemolysis activity was assessed by visualizing clear zones around the colony.

### Virulence studies in mice

To determine the residual virulence of the S-8*△clpP△apxIIC* mutant, various concentrations of *A. pleuropneumoniae* strains S-8 and S-8*△clpP△apxIIC* mutant were injected intraperitoneally into mice. One hundred and ten 6-week-old female BALB/c mice (Beijing Vital River Laboratory Animal Co., Ltd.) were randomly divided into eleven experimental groups (*n* = 10). Five experimental groups were inoculated with 100 μL of PBS containing the S-8*△clpP△apxIIC* mutant (1.0 × 10^8^ to 1.0 × 10^10^ CFU/mouse, Table 2). As a positive control, five experimental groups were inoculated with the wild-type strain S-8 (1.0 × 10^5^ to 1.0 × 10^7^ CFU/mouse, Table 2) using the identical method. Non-infected mice in the control group were inoculated with 100 μL of sterile PBS. After infection, mice were monitored twice daily for a 14-day period and humanely euthanized if moribund [22]. The 50% lethal dose (LD_50_) values of S-8 and S-8*△clpP△apxIIC* were calculated by Karber’s method [23].

**Table 2:** Virulence of S-8*△clpP△apxIIC* and the wild-type strain S-8 of *A. pleuropneumoniae* in mice

Strains	Challenge dose^*a*^ (CFU)	No. dead/No.tested	Value of LD50^*b*^ (CFU)	Fold attenuation^*c*^
S-8	1.0 × 10^7^	10/10	5.62 × 10^5^	1
	3.16 × 10^6^	9/10		
	1.0 × 10^6^	6/10		
	3.16 × 10^5^	5/10		
	1.0 × 10^5^	0/10		
S-8*△clpP△apxIIC*	1.0 × 10^10^	10/10	1.12 × 10^9^	1995
	3.16 × 10^9^	8/10		
	1.0 × 10^9^	5/10		
	3.16 × 10^8^	1/10		
	1.0 × 10^8^	0/10		
Control	PBS broth	0/10	-	-

^*a*^ Groups of ten BALB/c mice were given intraperitoneal injections of 100 μL of bacterial suspension containing various quantities of *A. pleuropneumoniae* strains. Survival was recorded for 14 days after infection

^*b*^ LD_50_ was calculated by the Karber’s method [23]

^*c*^ Fold attenuation was normalized to wild-type strain S-8

### Virulence studies in pigs

Twenty-five 8-week-old pigs were randomly assigned into five experimental groups (*n* = 5). The pigs in group 1 were inoculated with 1 × 10^7^ CFU of S-8*△clpP△apxIIC* via an intranasal (i.n.) route. The pigs in group 2 were injected with 1 × 10^7^ CFU of S-8 via the i.n. route. The pigs in group 3 were injected with 1 × 10^9^ CFU of S-8*△clpP△apxIIC* via the i.n. route. The pigs in group 4 were injected with 1 × 10^9^ CFU of S-8 via the i.n. route. The pigs in group 5, the control group, were inoculated with an equivalent amount of PBS via the i.n. route. The rectal temperature, appetite, respiratory rate, and lethargywere recorded daily for 14 days after inoculation as described previously [24]. Pigs that showed severe respiratory distress during the observation period were euthanized. All of surviving pigs were euthanized at day 14 post-challenge, and the lung lesions were examined and scored as described previously [25]. Briefly, the lung lesion was determined by divided the complete lung into seven lobes, each lobe was scored 1-5 by assessing the pneumonic area.

### Protection studies in pigs

Twenty 4-week-old pigs were randomly assigned into four experimental groups (*n* = 5). The pigs in group 1 and group 3 were immunized via an intramuscular (i.m.) route with 1 × 10^7^ CFU of S-8*△clpP△apxIIC* in 1 mL of PBS. The pigs in group 2 and group 4 were inoculated with 1 mL of PBS. The booster immunization was performed 21 days after the primary vaccination.

On day 14 following the booster immunization, the pigs in groups 1 and group 2 were challenged with 5.0 × 10^9^ CFU of *A. pleuropneumoniae* homologous serovar 7 (S-8) via the i.n. route. The pigs in groups 3 and group 4 were challenged with 5.0 × 10^8^ CFU of *A. pleuropneumoniae* heterologous serovar 5a (K17) via the i.n. route. After challenge, the pigs from each group were observed daily for clinical symptoms for 14-day period after challenge as previously described [24]. Pigs that showed severe respiratory distress during the observation period were euthanized. At day 14 post-challenge, all surviving pigs were euthanized and lung lesions were examined and scored as mentioned above [25].

#### Antibody measurements

Serum samples were collected from pigs in different groups before the first immunization (day 0), before the booster immunization (day 21) and before homologous or heterologous challenge (day 35). Antibodies against ApxII were examined using indirect ELISA as previously described [26]. 96-well plates were coated with 5 μg/mL of ApxII in 50 mM sodium carbonate buffer (pH 9.6) at 4 °C overnight. The wells were washed three times with PBST buffer (PBS supplemented with 0.05% Tween-20) and then blocked with PBS containing 5% bovine serum albumin at 37 °C for 1 h. Serum samples diluted in PBS were then added to the wells and incubated for 1 h at 37 °C. After the plates were washed, horseradish peroxidase (HRP)-conjugated goat anti-porcine IgG was diluted and added to the plates and incubated for 1 h at 37 °C. For determining the IgG isotypes, the sera were added to the S-8*△clpP△apxIIC*-coated plates and incubated with mouse anti-pig IgG1 Monoclonal Antibody (Clone K139 3C8, Thermo, United Kingdom) or mouse anti-pig IgG2 Monoclonal Antibody (Clone K68 Ig2, Thermo, United Kingdom), followed by HRP-conjugated goat anti-mouse IgG (Abcam, United Kingdom). After three washes, the substrate solution tetramethylbenzidine (TMB) and H_2_O_2_ were added to the wells and incubated for 15 min, and the reaction was stopped by the addition of 2 M sulfuric acid. The absorbance was measured at 450 nm using an ELISA reader. Each sample was tested in triplicate.

#### Determination of cytokines by ELISA

Serum samples at day 0, day 21, and day 35 were analyzed for swine gamma interferon (IFN-γ), interleukin 12 (IL-12) (R&D Systems, USA), and interleukin 4 (IL-4) (Invitrogen, USA) using ELISA kits performed according to the manufacturer’s instructions. Concentrations of swine IFN-γ, IL-12, and IL-4 in tested sera samples were determined by extrapolation to the linear portion of the standard curve, which was generated with supplied reference standards.

#### Bacterial loads analysis

Pigs from each group were necropsied immediately after euthanasia and lung tissues were aseptically collected. Samples were weighed, suspended in 1 mL PBS, and homogenized using a tissue homogenizer. The tissue homogenates were serially diluted with sterile PBS. Viable counts in serial dilutions of homogenates were determined following culture on TSA plates for 18 h at 37 °C. Identification of *A. pleuropneumoniae* was conducted by colony PCR assay and expressed as log_10_ CFU/g.

#### Statistical analysis

All statistical analyses were conducted using GraphPad Prism version 5.01 (GraphPad Software Inc., USA). Student’s *t*-test was used to evaluate the significance of the differences between multiple experimental groups. The data were expressed as the mean +/- standard deviation and values of *P* < 0.05 were considered to be significant.

## Results

### Construction of the S-8△clpP△apxIIC mutant strain

**Fig. 1:**
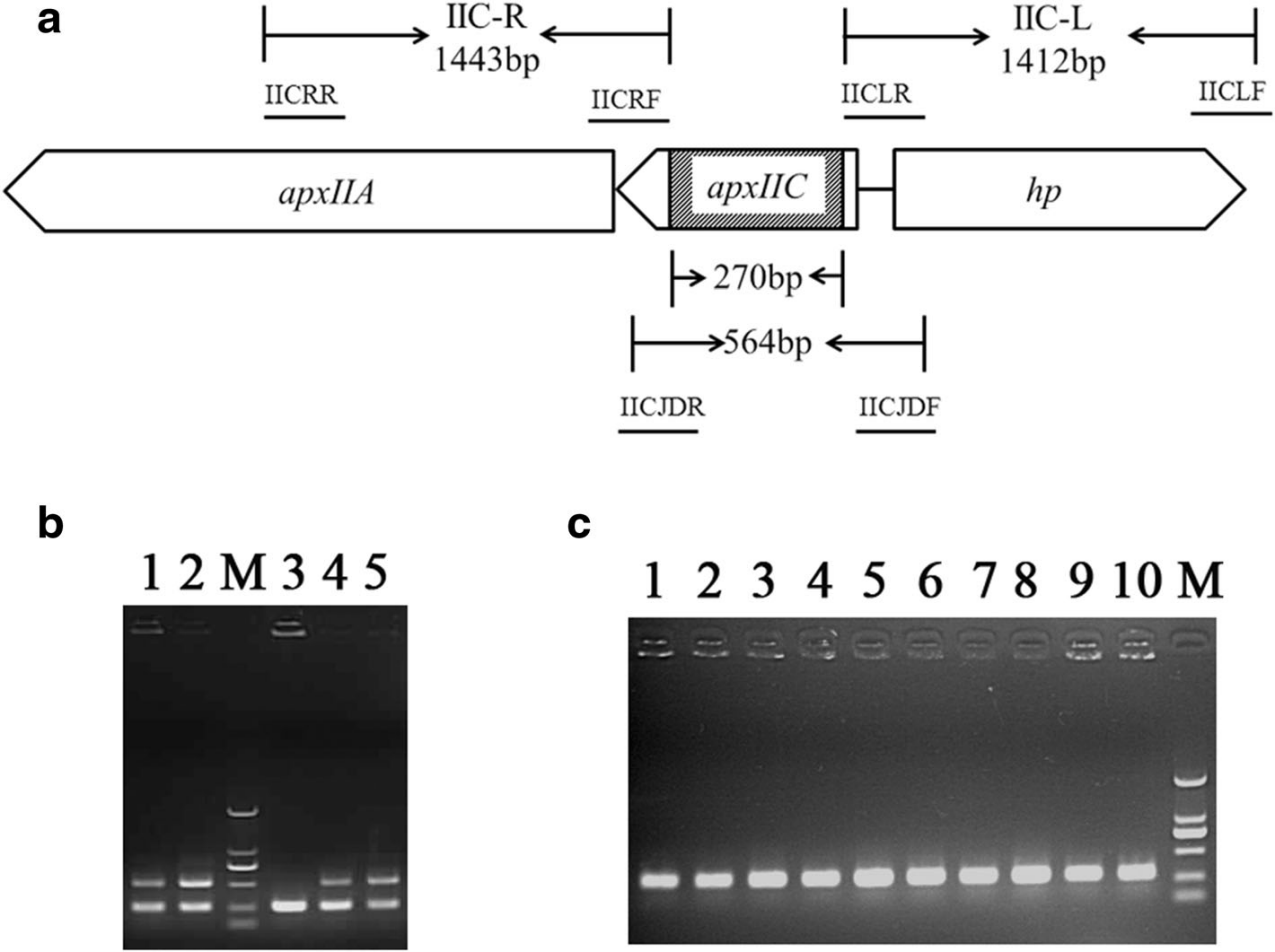
Characterization of the *A. pleuropneumoniae* mutant S-8*△clp△apxIIC*. **a** Schematic representation of the *A. pleuropneumoniae apxIIC* locus. Binding locations for the primers IICLF/IICLR and IICRF/IICRR used to amplify the two flanking regions (1412 bp and 1443 bp, respectively) of the *apxIIC* gene were shown in the schematic, and primers IICJDF/IICJDR were used to identify the S-8*△clp△apxIIC* mutant (294 bp) and S-8*△clp* mutant (564 bp). The shadowed domain represents a 270 bp in-frame deletion in the *apxIIC* gene, hp indicates the encoding gene of the hypothetical protein. **b** PCR identification of the S-8*△clp△apxIIC* mutant using the primers IICJDF/IICJDR. For lane 3, the identified S-8*△clp△apxIIC* mutant (294 bp); for other lanes, the single crossover mutant (564 bp and 294 bp). **c** PCR identification of the 10 passages of the S-8*△clp△apxIIC* mutant. For lanes 1–10, 10 successive passages of the S-8*△clp△apxIIC* mutant; for lane M, DL2000 DNA molecular marker (from top to bottom: 2000, 1000, 750, 500, 250, and 100 bp)

To construct the double-deletion mutant S-8*△clpP△apxIIC*, we deleted the *apxIIC* gene of *A. pleuropneumoniae* S-8*△clpP* mutant via the allelic exchange of the wild-type *apxIIC* gene with an unmarked, in-frame deletion lacking 270 bp of the *apxIIC* ORF (Fig. 1a and Fig. 1b). To test the stability of the in-frame deleted *apxIIC* gene in the genome of the *A. pleuropneumoniae* S-8*△clpP△apxIIC* mutant, a PCR assay was performed on the genomes of the mutant from 10 passages to detect a 294-bp DNA fragment characteristic of the in-frame deleted *apxIIC*. This fragment was observed in all 10 consecutive passages (Fig. 1c), suggesting a stable in-frame deletion in the *S-8△clpP△apxIIC* genome.

**Fig. 2:**
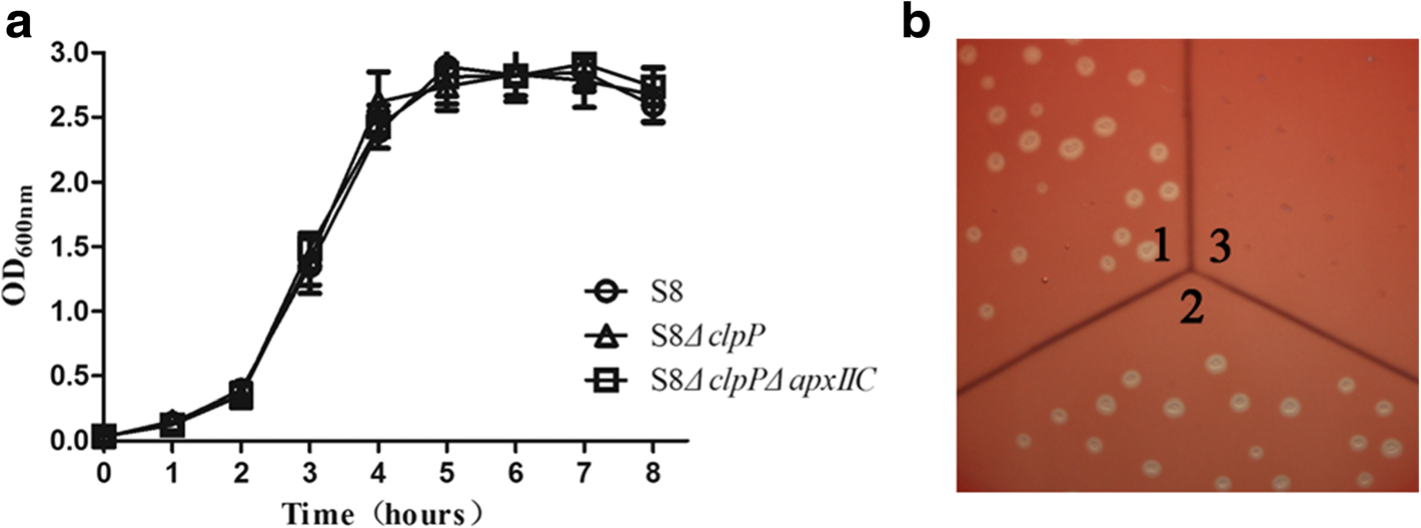
Growth characteristics and hemolytic activities of the *A. pleuropneumoniae* mutants. **a** The growth curves of the S-8, S-8*△clp* and S-8*△clp△apxIIC* strains. Overnight cultures were inoculated into fresh TSB and then incubated at 37 °C. Growth was monitored by OD600 at an interval of 1 h. **b** Hemolytic activity test for the S-8, S-8*△clp* and S-8*△clp△apxIIC* strains. Section 1, S-8; section 2, S-8*△clp*; section 3, S-8*△clp△apxIIC*

The growth curves of the S-8*△clpP* mutant, the S-8*△clpP△apxIIC* mutant, and the wild-type strain S-8 were similar at 37 °C (Fig. 2a). The hemolytic assay was examined in the wild-type strain S-8, the S-8*△clpP* mutant, and the S-8*△clpP△apxIIC* mutant. S-8 and the S-8*△clpP* mutant with the integrated *apxII* operon had hemolytic activity, as shown by clear zones around the colonies (Fig. 2b). However, the clear zones were absent in the S-8*△clpP△apxIIC* mutant, the result of deletion of the *apxIIC* gene rendered it unable to activate ApxII toxin, and thus lacking hemolytic activity.

### Virulence of the S-8△clpP and S-8△clpP△apxIIC mutants in mice

The attenuation of virulence was investigated by determining the LD_50_ values of the S-8*△clpP△apxIIC* mutant, and the wild-type strain S-8 in BALB/c mice. The data showed LD_50_ values of 5.62 × 10^5^ CFU per mouse for the wild-type strain S-8, and 1.12 × 10^9^ CFU per mouse for the S-8*△clpP△apxIIC* mutant. Compared to the wild-type strain S-8, the S-8*△clpP△apxIIC* mutant was attenuated by approximately 1195-fold in mice. LD_50_ values showed that the S-8*△clpP△apxIIC* mutant was highly attenuated.

### Virulence of the S-8△clpP△apxIIC mutant in pigs

**Fig. 3:**
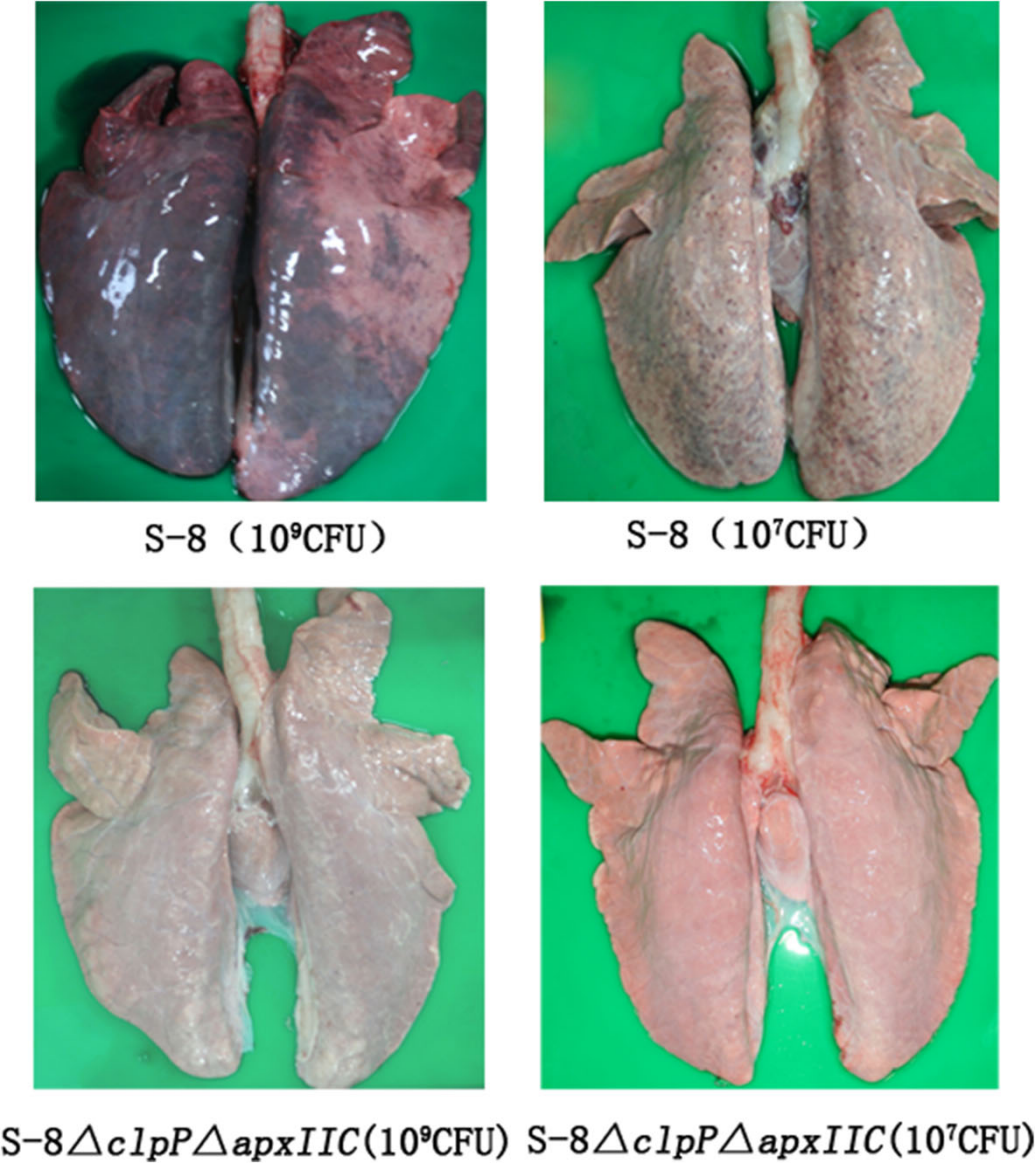
Pathological changes of the lungs of pigs infected with *A. pleuropneumoniae*. Groups of pigs were inoculated with different doses of S-8 and S-8*△clp△apxIIC*. At 14 days post-infection, all pigs were euthanized and lungs were collected and subjected to pathological examination

The results of the safety study on the S-8*△clpP△apxIIC* mutant in pigs are listed in Table 3. Two of five pigs inoculated with 1.0 × 10^9^ CFU of the S-8*△clpP△apxIIC* mutant via the i.n. route showed a slight increase in rectal temperatures (40.1 °C < body temperatures < 40.3 °C) after 8–20 h post-infection and exhibited only mild clinical symptoms of porcine pleuropneumonia, such as decreased appetite. However, all of these pigs recovered quickly in 24 h and were in good health afterward. All of the five pigs inoculated with 1.0 × 10^7^ CFU of the S-8*△clpP△apxIIC* via the i.n. route exhibited no clinical signs of porcine pleuropneumonia. Compared to the groups inoculated with the S-8*△clpP△apxIIC* mutant, the groups inoculated with the S-8 strain exhibited more severe clinical symptoms of porcine pleuropneumonia. Three of five pigs which were inoculated with 1.0 × 10^9^ CFU of S-8 were euthanized because of severe clinical symptoms. The lesions in their lungs were severe with massive hemorrhages and fibrinous inflammation was observed. Compared to serious lung lesions of pigs in the S-8-inoculated groups, there are no or few lung lesions in the pigs of the S-8*△clpP△apxIIC*-inoculated groups (Fig. 3). The average lung lesion scores were 16.2 for 1.0 × 10^7^ CFU and 21.8 for 1.0 × 10^9^ CFU of S-8 challenge. However, the groups inoculated with S-8*△clpP△apxIIC* showed significantly lower lung lesion scores, with 1.8 and 3.0 following challenge with 1.0 × 10^7^ CFU or 1.0 × 10^9^ CFU, respectively (Table 3).

**Table 3:** Virulence of the *A. pleuropneumoniae* S-8*△clpP△apxIIC* mutant in pigs

Group	Strain for challenge	Challenge dose^*a*^ (CFU)	Temperature (°C) ^*b*^	Appetite^*c*^	Lethargy^*c*^	Dyspnea^*c*^	Lung lesion score ^*d*^
1	S-8*△clpP△apxIIC*	1.0 × 10^7^	39.2 ± 0.3	0.2 ± 0.4^*^	0.1 ± 0.2^**^	0.2 ± 0.2^**^	1.8 ± 3.0^**^
2	S-8	1.0 × 10^7^	40.1 ± 0.4	1.8 ± 1.3	1.7 ± 0.7	1.9 ± 0.7	16.2 ± 8.5
3	S-8*△clpP△apxIIC*	1.0 × 10^9^	39.4 ± 0.5	0.4 ± 0.5^*^	0.3 ± 0.3^**^	0.4 ± 0.4^**^	3.0 ± 2.2^**^
4	S-8	1.0 × 10^9^	40.2 ± 0.6	2.4 ± 1.6	2.2 ± 0.6	2.5 ± 0.5	21.8 ± 5.8
5	PBS	0	39.4 ± 0.3	0	0	0	0

^*a*^ Groups of five pigs were given intranasal inoculations with 1 mL of bacterial suspension containing various quantities of *A. pleuropneumoniae* strains. Survival was recorded for 14 days after infection

^*b*^ The average temperature value for each piglet during the observation period was calculated and the mean temperature value for each group was determined

^*c*^ Clinical signs were scored as described by Jolie et al [24]. Appetite was scored as follows: 0, did eat; and 1, did not eat. The total score equaled the number of 12-h periods of not eating over the 36-h observation period. Lethargy was scored as follows: 0, normal; 1, slight inactivity; 2, moderate; and 3, severe. Dyspnea was scored as follows: 0, normal; 1, slight; 2, moderate; and 3, severe. The final scores were obtained from the average of all of the data within the observation time and are expressed as arithmetic means ± SD; *, significance at a *P* value of <0.05; **, significance at a *P* value of <0.01

^*d*^ The lung lesion score was determined as described by Hannan et al [25]

### Immune response of pigs to the S-8△clpP△apxIIC mutant

**Fig. 4:**
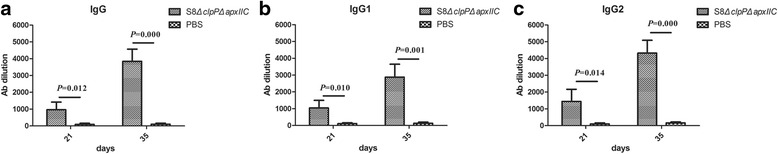
Levels of IgG antibody (**a**), IgG1 (**b**), and IgG2 (**c**) in the sera of piglets. Blood samples were collected prior to and following immunization on days 0, 21, and 35 from the S-8*△clp△apxIIC* immunized groups and PBS control groups, and the antibody responses were determined by indirect ELISA. The results are expressed as the means ± SD

Serum samples from pigs of each group were obtained from blood via anterior vena cava venipuncture. Figure 4a showed a significant increase in antibody titers in pigs immunized with S-8*△clpP△apxIIC* on days 21 and 35, however, no antibody was detected in the PBS control groups. The IgG isotype was determined to check the specific antibody types against S-8*△clpP△apxIIC*. The levels of isotypes IgG1 and IgG2 in the immunized groups were significantly higher (*P* < 0.01) than that of PBS control groups (Fig. 4b, c).

**Fig. 5:**
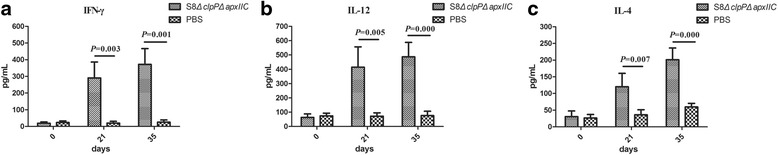
Levels of IFN-γ, IL-12, and IL-4 in the sera of piglets. Blood samples were collected prior to and following immunization on days 0, 21, and 35 from the S-8*△clp△apxIIC* immunized groups and PBS control groups, and the levels of IFN-γ (**a**), IL-12 (**b**), and IL-4 (**c**) were determined by commercial kit. The results are expressed as the means ± SD

Levels of IFN-γ in sera from S-8*△clpP△apxIIC* immunization groups were significantly higher than those of PBS control groups on days 21 and 35 (*P* < 0.01) (Fig. 5a). Levels of IL-12 in the S-8*△clpP△apxIIC* immunization groups were also significantly higher than those of control groups during the observation period (*P* < 0.01) (Fig. 5b). Both the IFN-γ and IL-12 concentrations in sera from S-8*△clpP△apxIIC* immunized animals increased substantially on day 21 and exhibited a smaller increase on day 35. While the IL-4 concentrations in sera from S-8*△clpP△apxIIC* immunized groups exhibited an approximately equal increase on day 21 and day 35, higher than in sera from the PBS control groups (*P* < 0.01) (Fig. 5c).

### Protective efficacy in pigs

**Fig. 6:**
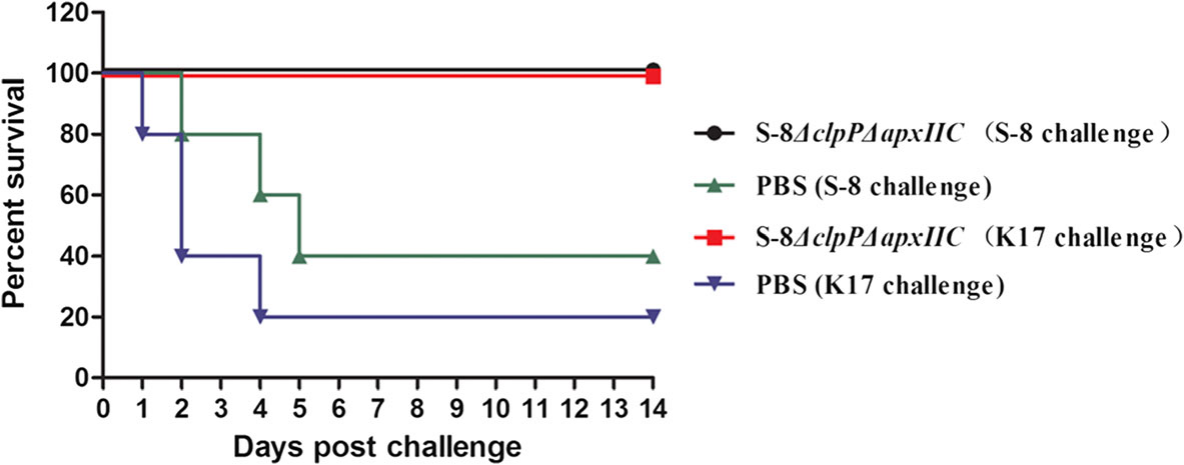
Survival of pigs following intranasal challenge with *A. pleuropneumoniae* strains S-8 or K17

The protective efficacy of the S-8*△clpP△apxIIC* mutant against lethal challenge with *A. pleuropneumoniae* serovar 7 S-8 or serovar 5a K17 in pigs was evaluated in terms of body temperature, clinical signs, lung lesions, and survival rate. The results are summarized in Fig. 6 and Table 4. Pigs in the S-8*△clpP△apxIIC*-immunized groups showed slight or no lethargy, anorexia or dyspnea after challenge with *A. pleuropneumoniae* serovar 5a or serovar 7. Four immunized pigs had a transient increased body temperature (<40.3 °C) on day 0 upon challenge with *A. pleuropneumoniae* S-8 or K17 but recovered afterward. During the 14-day observation period, all immunized pigs survived with clinical symptoms ranging from none to only mild. All of the pigs in the PBS control groups developed anorexia, increased respiratory rate, and depression after challenge with *A. pleuropneumoniae* S-8 or K17. The average body temperature increased (41.2 °C) for at least 3 days. Four of ten pigs showed severe respiratory distress within 48 h and were euthanized. Three pigs subsequently exhibited severe respiratory distress during the next four days and were euthanized. Only two pigs challenged with S-8 and one pig challenged with K17 in the control groups survived over the 14-day observation period.

**Table 4:** Protective efficacy of the S-8*△clp△apxIIC* mutant against *A. pleuropneumoniae* S-8 and K17 challenge in pigs

Group	Immunogen	Strain for challenge	Temperature (°C) ^*a*^	Appetite^*b*^	Lethargy^*b*^	Dyspnea^*b*^	Lung lesion score^*c*^
1	S-8*△clpP△apxIIC*	S-8	39.4 ± 0.2	0.2 ± 0.3^*^	0.2 ± 0.3^**^	0.1 ± 0.2^**^	1.4 ± 2.2^*^
2	PBS	S-8	40.3 ± 0.5	2.4 ± 1.1	2.0 ± 0.6	2.3 ± 0.6	20.2 ± 11.9
3	S-8*△clpP△apxIIC*	K17	39.6 ± 0.3	0.3 ± 0.4^*^	0.3 ± 0.4^**^	0.4 ± 0.4^**^	1.7 ± 2.5^**^
4	PBS	K17	40.5 ± 0.4	2.6 ± 1.7	2.3 ± 0.5	2.6 ± 0.4	22.8 ± 9.3

^*a*^ The average temperature value for each piglet during the observation period after challenge was calculated and the mean temperature value for each group was determined

^*b*^ Clinical signs were scored as described by Jolie et al [24]. Appetite was scored as follows: 0, did eat; and 1, did not eat. The total score equaled the number of 12-h periods of not eating over the 36-h observation period. Lethargy was scored as follows: 0, normal; 1, slight inactivity; 2, moderate; and 3, severe. Dyspnea was scored as follows: 0, normal; 1, slight; 2, moderate; and 3, severe. The final scores were obtained from the average of all of the data within the observation time and are expressed as arithmetic means ± SD; *, significance at a *P* value of <0.05; **, significance at a *P* value of <0.01

^*c*^ The lung lesion score was determined as described by Hannan et al [25]

At necropsy, the pigs in the PBS control groups showed severe lung lesions and pleuritis. Hemorrhage and fibrinous exudation on the lung and pleura were found in these pigs. The average lung lesion scores were 20.2 and 22.8 for challenge with *A. pleuropneumoniae* S-8 or K17, respectively. However, in comparison to the PBS control groups, the S-8*△clpP△apxIIC*-immunized groups showed significantly lower lung lesion scores of 1.4 and 1.8 for challenge with *A. pleuropneumoniae* S-8 or K17, respectively.

#### Bacteriological analysis of tissue homogenates

**Fig. 7:**
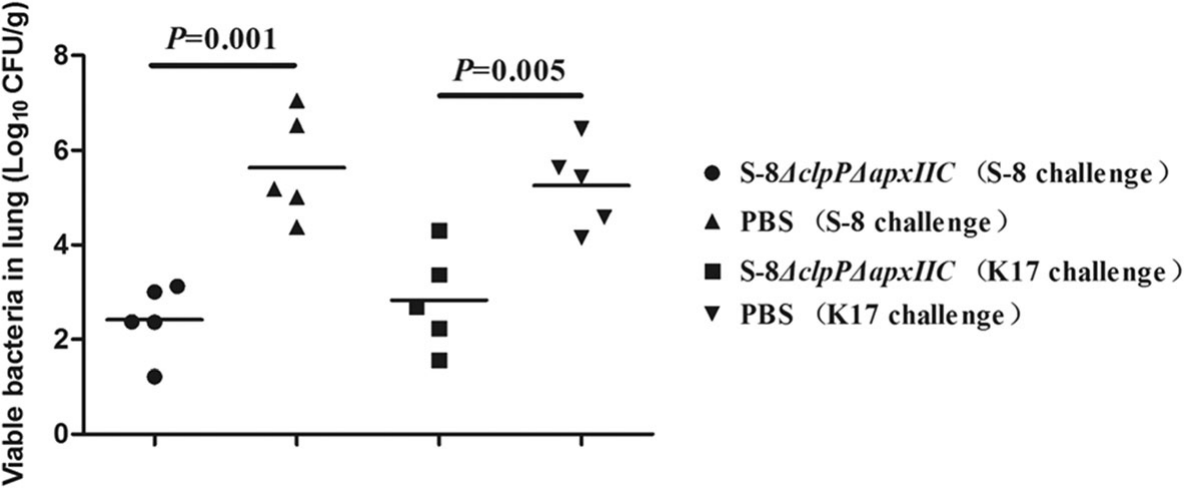
Bacterial loads in lung homogenates after challenge with *A. pleuropneumoniae* strains S-8 or K17. The logarithm value (Log_10_) of the CFU in each gram of tissue sample was recorded. The immunized groups were vaccinated with S-8*△clp△apxIIC* and the PBS control groups were injected with PBS. The results are expressed as the means ± SD

Bacterial loads in lung homogenates were counted after challenge with *A. pleuropneumoniae* S-8 or K17 (Fig. 7). The numbers of CFUs recovered from the homogenized lung tissues in S-8*△clpP△apxIIC* immunized groups were significantly lower (*P* < 0.01) than those of PBS control groups.

## Discussion

With the growing emergence of drug resistance and the problem of antibiotic residues, vaccination becomes the most effective method of preventing *A. pleuropneumoniae* infection [7, 11]. Previous studies found that pigs surviving natural infection of *A. pleuropneumoniae* could be fully protected against homologous strain and partially against heterologous serovars, suggesting that live bacteria likely induced in vivo expression of protective antigens and conferred cross-protection [11]. Thus, an attenuated live vaccine is widely acknowledged as an ideal approach for vaccination against porcine pleuropneumonia.

The ideal live vaccine of porcine pleuropneumonia should be low virulent and cause minimum lung lesions [11]. Our previous study constructed S-8*△clpP*, an *A. pleuropneumoniae clpP* gene deletion mutant, and illustrated the important function of the ClpP protease in the stress response and biofilm formation of *A. pleuropneumoniae*, suggesting a putative role for ClpP protease in the virulence regulation [17]. Afterwards, the virulence of S-8*△clpP* was determined using the BALB/c mouse infection model. The finding that the S-8*△clpP* moderately attenuated by approximately 71-fold (data not shown) was unexpected as a previous study had found that *clpP* deletion strains of *S. typhimurium* and *S. enteritidis* were attenuated by approximately 10,000-fold [16]. Thus, in this study, we further deleted the *apxIIC* gene that encodes the ApxII activating protein, and rendered the double-deletion mutant S-8*△clpP△apxIIC* secreting unactivated ApxII toxins but with complete antigenicity [18]. Compared to the wild-type strain, S-8*△clpP△apxIIC* was greatly attenuated by approximately 1195-fold. We next evaluated the virulence of S-8*△clpP△apxIIC* in pigs via intranasal inoculation, with the results showing that pigs inoculated with 1.0 × 10^7^ CFU of the S-8*△clpP* mutant displayed no clinical signs of porcine pleuropneumonia but exhibited only transient depression when inoculated with 1.0 × 10^9^ CFU. Moreover, there are no or little lung lesions in the pigs of the S-8*△clpP△apxIIC*-inoculated group, which showed that the S-8*△clpP△apxIIC* mutant was adequately attenuated and has almost no detrimental effects on pigs that remained healthy throughout the experiment.

An essential characteristic for an effective attenuated live vaccine is that the strain should remain highly immunogenic [11]. Pigs vaccinated with the *A. pleuropneumoniae* S-8*△clpP△apxIIC* mutant exhibited a significantly increased ApxII-specific IgG Ab response compared to pigs injected with PBS. Interestingly, both *A. pleuropneumoniae*-specific IgG1 and IgG2 titers increased following the first immunization and booster immunization. The production of IgG isotypes in pig is elicited by type 1 (IFN-γ, IL-12) and type 2 (IL-4) cytokines, which lead the responses to a cell-mediated or antibody-mediated immune response [27]. In pigs, IgG2 is linked to the production of IFN-γ and IL-12 and correlates with the Th1 response [27, 28]. Conversely, the production of the specific IgG1 antibody partially relies on the presence of the Th2 cytokine IL-4 [29]. In this study, we also found that on day 35, the levels of IFN-γ, IL-12, and IL-4 were significantly higher in sera from S-8*△clpP△apxIIC* immunized pigs than those in sera from PBS control groups, but IL-4 concentrations were lower than IFN-γ and IL-12 concentrations in sera from the immunization groups. These data suggested that immunization with *A. pleuropneumoniae* S-8*△clpP△apxIIC* generated a slight bias towards the Th1-type immune response. However, the IgG1 titers and IL-4 concentrations in the S-8*△clpP△apxIIC* immunized pigs were still much higher than those in the PBS control groups. These data showed that the Th2-type immune response also plays a partial role in immunization with live *A. pleuropneumoniae* S-8*△clpP△apxIIC*. Unlike the other *A. pleuropneumoniae* live attenuated mutant that is significantly biased toward a Th1-type immune response [30], S-8*△clpP△apxIIC* generated a more balanced and broader immune response.

Cross-protection is a crucial characteristic that is important to achieve widespread use of a vaccine. Our findings demonstrated that immunization with the S-8*△clpP△apxIIC* mutant could induce acquired immunity and confer a marked resistance against the lethal challenge with *A. pleuropneumoniae* virulent homologous strain S-8 and heterologous serovar 5a. Although S-8*△clpP△apxIIC* exhibited good immune protection as a live vaccine, a few pigs after challenge still had few pathological lesions. As it is unlikely that the multiple-gene deleted mutant can revert back to the wild-type genotype, we will further delete other important virulence genes of *A. pleuropneumoniae* in our future studies and construct a multiple-gene deleted mutant as a safe, attenuated live vaccine to prevent and control *A. pleuropneumoniae* infection.

## Conclusion

In conclusion, data presented in this study indicated that the immunizations with the candidate vaccine S-8*△clpP△apxIIC* were safe in pigs; and conferred efficient protection against the homologous or heterologous serovar infection. Overall, the S-8*△clpP△apxIIC* mutant of *A. pleuropneumoniae* has the potential as a novel live attenuated vaccine against porcine pleuropneumonia, although further trials are needed.

## Abbreviations

*A. pleuropneumoniae*: *Actinobacillus pleuropneumoniae*
ATP: Adenosine triphosphate
CFU: Colony-forming unit
ELISA: Enzyme-linked immunosorbent assay
IFN-γ: Gamma interferon
IgG: Immunoglobulin G
IL-12: Interleukin 12
IL-4: Interleukin 4
LB: Luria-Bertani
LD_50_: Lethal dose 50% value
PCR: Polymerase chain reaction
*S. enteritidis*: *Salmonella enteritidis*
*S. typhimurium*: *Salmonella typhimurium*
SOE PCR: Single-overlap extension polymerase chain reaction
TSA: Tryptic soy agar
TSB: Tryptic soy broth
